# Surgery Plus Chemotherapy Versus Surgery Alone for Limited-Stage Small-Cell Lung Cancer: A Population-Based Survival Outcome Analysis

**DOI:** 10.3389/fonc.2021.676598

**Published:** 2021-05-17

**Authors:** Pingting Ye, Zhuolin Guo, Yanfei Zhang, Chunyan Dong, Ming Li

**Affiliations:** ^1^ Department of Respiratory Medicine, Shanghai 10th People's Hospital, Tongji University School of Medicine, Shanghai, China; ^2^ Department of Oncology, Shanghai East Hospital, Tongji University School of Medicine, Shanghai, China; ^3^ Department of Dermatology, Shanghai Tenth People’s Hospital, Tongji University School of Medicine, Shanghai, China

**Keywords:** small-cell lung cancer, surgery, chemotherapy, overall survival, Surveillance, Epidemiology and End Results (SEER)

## Abstract

**Introduction:**

For patients with limited-stage small-cell lung cancer (LS-SCLC), effective treatment methods still remain a clinical challenge. The aim of this study is to evaluate the survival outcome of surgery plus chemotherapy *vs.* surgery alone in patients with LS-SCLC.

**Methods:**

LS-SCLC patients selected from the Surveillance, Epidemiology and End Results (SEER) database diagnosed between January 1, 2004, and December 31, 2015. Comparison of overall survival (OS) and cancer-specific survival (CSS) between two groups performed propensity score matching (PSM), inverse probability of treatment weight (IPTW), and overlap weighting analysis.

**Results:**

Of the 477 LS-SCLC patients identified from the SEER database between 2004 and 2015, 262 (54.9%) received surgery-plus-chemotherapy treatment and the others received surgery-alone treatment. Univariate and multivariate analyses showed that treatment option (*P*< 0.001), tumor location (*P*= 0.02) and AJCC stage (*P*< 0.001) were independent prognostic predictors of OS in LS-SCLC patients. Median OS was 35 months in surgery-plus-chemotherapy group *vs.* 23 months in surgery-alone group. Survival analysis showed that surgery plus chemotherapy offered significantly improved OS as compared with surgery-alone treatment before and after IPTW, PSM and overlap weighting method (all *P*< 0.05). According to AJCC stage stratification, OS of the unmatched patients with stage I (*P*= 0.049) and II (*P*= 0.001) SCLC who received surgery-plus-chemotherapy treatment was significantly better than that of surgery-alone patients.

**Conclusions:**

This cohort study showed that surgery plus chemotherapy was associated with longer survival time than surgery alone in LS-SCLC patients, especially in those with stage I and II SCLC. Further prospective studies are required to confirm our conclusions.

## Introduction

Small cell lung cancer (SCLC), a type of clinically aggressive neuroendocrine malignancy, accounts for about 13.1% of all types of lung cancer, characterized by rapid growth and early development of extensive metastasis, leading to a generally poor prognosis of patients (1, 2). For the patients with limited-stage SCLC (LS-SCLC), therefore, effective treatment methods remain a critical and essential goal.

According to the National Comprehensive Cancer Network (NCCN) guidelines (3), the primary treatment for SCLC at early stages is surgery, including mediastinal lymphadenectomy or sampling following pathologic mediastinal staging. Because of rapid progression and early occurrence of blood-bone and lymph metastasis, local treatment alone may not effectively control recurrence (4, 5). Thus, systemic treatment should be considered for LS-SCLC. Currently, adjuvant chemotherapy following surgical resection is recommended as the standard treatment strategy for patients with stage II and IIIA NSCLC, but not for patients with stage I (6). However, there is no retrospective cohort study to answer the question whether this therapeutic strategy is also applicable to LS-SCLC. In the present study, the aim is to further evaluate the survival outcome of LS-SCLC patients who received surgery-plus-chemotherapy treatment *vs.* surgery-alone treatment by acquiring data from the Surveillance, Epidemiology and End Results (SEER) database in stage I–IIIA SCLC patients diagnosed between 2004 and 2015.

## Methods

### Data Source

Using SEER database from the National Cancer Institute (NCI), we collected data from January 1, 2004, to December 31, 2015. As a population-based cancer registry, the SEER Program was initiated in the USA in 1973, covering about 28% of the country’s population (7). The patient sample of this study was selected from publicly available de-identified data in the NCI SEER 18 Registries, which was allowed to be used in relevant medical research and deemed exempt from institutional review board oversight. In addition, the annual follow-up rate for all patients diagnosed with cancer in the past five years is 90%.

### Patient Selection

**Figure 1:**
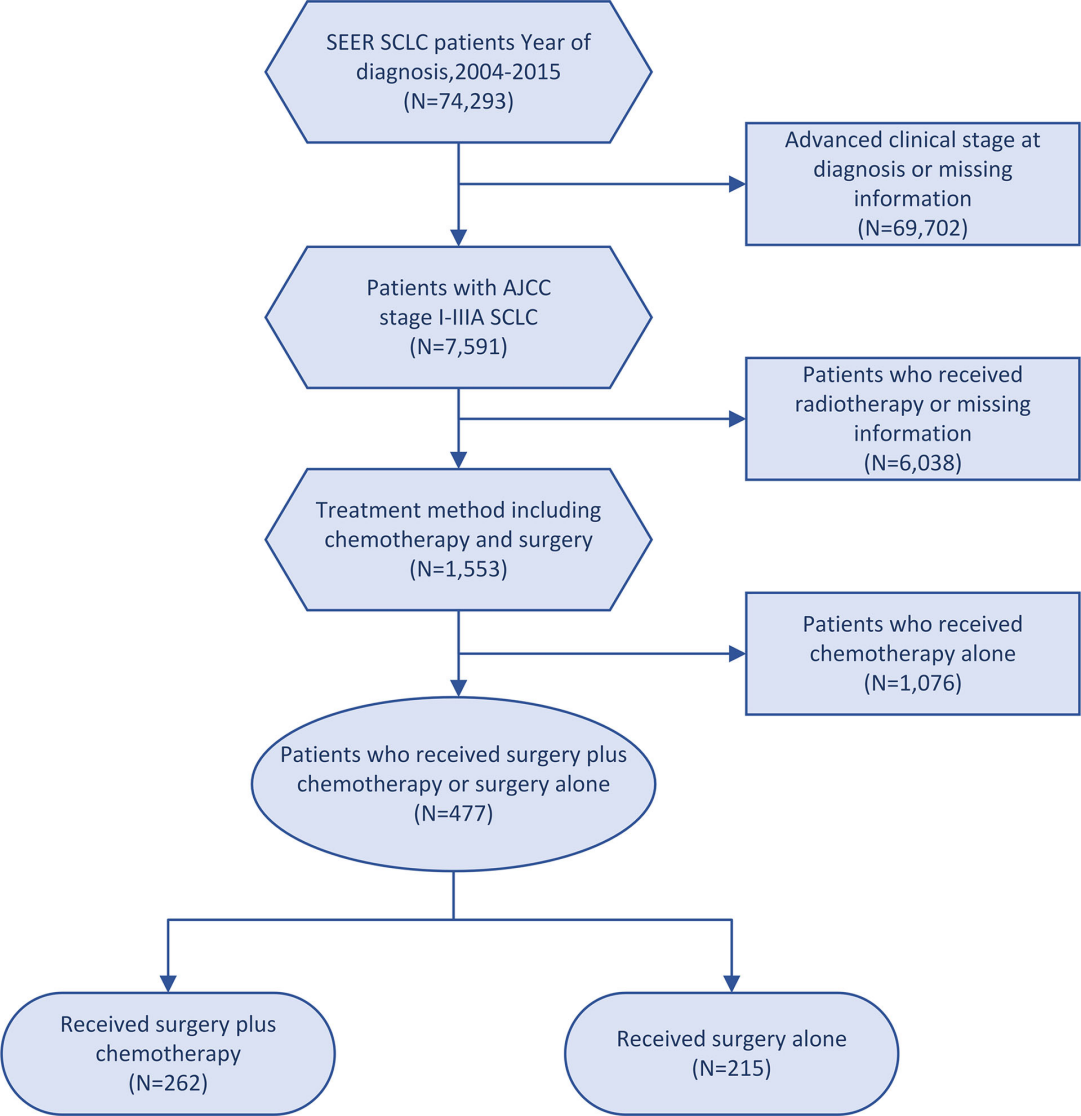
Flowchart of patient selection steps.

This cohort study included patients diagnosed with SCLC from January 2004 to December 2015. The inclusion criteria were as follows: (1) patients with stage I-IIIA disease according to the American Joint Committee on Cancer (AJCC) 7^th^ edition; and (2) patients undergoing surgery-plus-chemotherapy or surgery-alone treatment. The exclusion criteria were as follows: (1) patients receiving radiotherapy; (2) patients with missing information regarding age, sex, race, tumor size, AJCC stage, laterality, tumor location and differentiated grade. Pathological staging was performed on the patients who underwent surgery. Finally, 477 patients recruited in this study, according to the treatment modality, were divided into a surgery-plus-chemotherapy group and a surgery-alone group (**Figure 1**). Complete information on patients was obtained from the SEER database.

### Covariates

The baseline covariates included race, sex, age of diagnosis, geographic region, tumor location, AJCC stage, differentiated grade, laterality, tumor size, median household income, insurance status, marital status and high school education based on the patient post code. The SEER data dictionary provided a comprehensive description of all included covariates for reference.

### Statistical Analysis

Chi-square and Wilcoxon rank-sum tests were applied to assess the correlations between different therapy methods and all the above-mentioned baseline covariates. A multivariable logistic regression model was used to estimate receipt of chemotherapy-plus-surgery treatment. Univariable and multivariable Cox proportional hazards models were performed for overall survival (OS) and cause-specific survival (CSS) in both treatment groups and adjusted all baseline covariates. To overcome the impact of patient selection bias, propensity score matching (PSM) method was used to balance possible confounding factors. All the covariates in our study were matched between the two groups. Additionally, three PS models (PSM, IPTW and overlap weighting method) were used in our study. Patients in the two groups were matched with a ratio of 1:1 (R package “MatchIt”). The IPTWs and overlap weights were calculated by using R package “PSW” with the covariates in the previous final PS model. For the PS models of OS and CSS, Kaplan-Meier methodology with log-rank test was used for all patients in this study. Next, we selected the two groups of patients based on the covariates into subgroups and conducted stratified analyses in a similar manner as described previously. Statistical analyses were performed using R version 4.0.3 (R Foundation for Statistical Computing, Vienna, Austria) and IBM SPSS Statistics 23.0 (IBM Corp., Armonk, NY, USA). Two-sided *P* values < 0.05 were considered statistically significant.

## Results

### Patient Characteristics

This study enrolled 477 patients fulfilled the eligibility criteria. **Table 1** shows the baseline characteristics of all patients extracted from the SEER database. The median age at diagnosis was 68 years (range: 31-89 years). Among these patients, the proportion of women was slightly greater (52.8%) than that of men population (47.2%). Most patients (91.0%) were white, 6.3% were black, and 2.7% were others. Most patients were in the east of the US (275/57.7%) and diagnosed with AJCC stage I (320/67.1%). Interestingly, all patients who received surgery were diagnosed after 2008 in our study. Of the 477 patients, 215 (45.0%) received surgery alone and the other 262 received surgery plus chemotherapy between 2004 and 2015. As shown in **Table 1**, significant differences were found in age of diagnosis (*P*= 0.002), differentiated grade (*P*= 0.01) and marital status (*P*= 0.02) between the two groups in the unadjusted cohorts. There was no statistically significant difference in race, sex, age of diagnosis, geographic region, tumor location, AJCC stage, differentiated grade, laterality, tumor size, insurance status, marital status, high school education, and median household income (all *P* > 0.05) between the two groups after matching (**Supplementary Figure 1**).

**Table 1:** Demographic and clinical characteristics of patients with small cell lung cancer.

Characteristics	No. (%)										
	Before propensity score matching			After propensity score matching			Standardized Difference				
	Chemotherapy Plus Surgery (n=262)	Surgery Alone (n=215)	*P*	Chemotherapy Plus Surgery (n=170)	Surgery Alone(n=170)	*P*	Unmatched	Matched	IPTW	Overlap Weighting	
Race			0.29			0.79	0.143	0.075	0.017	<0.001	
White	243(92.7)	191(88.8)		157(92.4)	154(90.6)						
Black	14(5.3)	16(7.4)		9(5.3)	10(5.9)						
Others	5(1.9)	8(3.3)		4(2.4)	6(3.5)						
Sex			0.99			>0.99	0.01	0.012	0.024	<0.001	
Male	123(46.9)	102(47.4)		79(46.5)	78(45.9)						
Female	139(53.1)	113(52.6)		91(53.5)	92(54.1)						
Age of diagnosis			0.002			0.96	0.381	0.089	0.028	<0.001	
≤44	1(0.4)	1(0.5)		1(0.6)	1(0.6)					
45-54	11(4.2)	13(6.0)		10(5.9)	8(4.7)					
55-64	57(21.8)	54(25.1)		39(22.9)	38(22.09)					
65-74	139(53.1)	76(35.3)		73(42.9)	68(40.0)					
≥75	54(20.6)	71(33.0)		47(27.6)	51(30.0)					
Region			0.06			0.97	0.254	0.052	0.009	<0.001	
East	156(59.5)	119(55.3)		95(55.9)	95(55.9)					
Northwest or West	63(24.0)	72(33.5)		54(31.8)	54(31.8)					
North	33(12.6)	21(9.8)		19(11.2)	18(10.6)					
Southwest	10(3.8)	3(1.4)		2(1.2)	3(1.8)					
Primary labeled			0.56			0.97	0.16	0.076	0.026	<0.001	
Upper lobe	158(60.3)	140(65.1)		112(65.9)	112(65.9)					
Middle lobe	16(6.1)	9(4.2)		8(4.7)	8(4.7)					
Lower	80(30.5)	58(27.0)		43(25.3)	45(26.5)					
Nos	6(2.3)	4(1.9)		5(2.9)	4(2.4)					
Overlapping	2(0.8)	4(1.9)		2(1.2)	1(0.6)					
AJCC 7^th^			0.16			0.68	0.18	0.095	0.052	<0.001	
stage I	170(64.9)	150(69.8)		114(67.1)	115(67.6)					
stage II	42(16.0)	38(17.7)		28(16.5)	32(18.8)					
stage III	50(19.1)	27(12.6)		28(16.5)	23(13.5)					
Grade			0.01			0.98	0.33	0.07	0.024	<0.001	
I	2(0.8)	6(2.8)		2(1.2)	3(1.8)					
II	4(1.5)	11(5.1)		4(2.4)	3(1.8)					
III	74(28.2)	75(34.9)		57(33.5)	57(33.5)					
Undifferentiated	87(33.2)	65(30.2)		52(30.6)	54(31.8)					
Unknown	95(36.3)	58(27.0)		55(32.4)	53(31.2)					
Laterality			0.86			0.74	0.025	0.048	0.042	<0.001	
Right	162(60.3)	127(59.1)		104(61.2)	100(58.8)					
Left	104(39.7)	88(40.9)		66(38.8)	70(41.2)					
Tumor size			0.13			>0.99	0.221	0.022	0.045	<0.001	
≤1cm	21(8.0)	25(11.6)		14(8.2)	15(8.8)					
1-2cm	104(39.7)	94(43.7)		76(44.7)	75(44.1)					
2-3cm	75(28.6)	43(20.0)		36(21.2)	36(21.2)					
>3cm	62(23.7)	53(24.7)		44(25.9)	44(25.9)					
Insurance Recode			0.42			0.95	0.151	0.063	0.051	<0.001	
Medicaid	34(13.0)	30(14.0)		22(12.9)	22(12.9)					
Uninsured	2(0.8)	4(1.9)		2(1.2)	1(0.6)					
Unknown	1(0.4)	3(1.4)		1(0.6)	1(0.6)					
Insured	225(85.9)	178(82.8)		145(85.3)	146(85.9)					
Marital status			0.02			0.99	0.309	0.064	0.026	<0.001	
Married	150(57.3)	104(48.4)		90(52.9)	86(50.6)					
Single	16(6.1)	31(14.4)		16(9.4)	17(10.0)					
Divorced	40(15.3)	31(14.4)		28(16.5)	27(15.9)					
Widowed	47(17.9)	37(17.2)		27(15.9)	30(17.6)					
Unknown	9(3.4)	12(5.6)		9(5.3)	10(5.9)					
Education			0.29			0.95	0.179	0.065	0.053	<0.001
<7	40(15.3)	36(16.7)		25(14.7)	29(17.1)					
7-12	110(42.0)	74(34.4)		60(35.3)	58(34.1)					
12-20	71(27.1)	60(27.9)		51(30.0)	50(29.4)					
>21	41(15.6)	45(20.9)		34(20.0)	33(19.4)					
Median household income (dollar, in tens)			0.68			0.89	0.112	0.087	0.05	<0.001
<38000	18(6.9)	20(9.3)		14(8.2)	16(9.4)					
38000-47999	46(17.6)	38(17.7)		31(18.2)	30(17.6)					
48000-62999	106(40.5)	78(36.3)		64(37.6)	58(34.1)					
>63000	92(35.1)	79(36.7)		61(35.9)	66(38.8)					

IPTW, inverse probability of treatment weight.

### Multivariable Logistic Regression Analysis, Univariate Analysis and Multivariate Analysis

For all covariates at baseline, no significant difference was found between the two groups *via* multivariable logistic regression analysis, except for the marital status (OR, 3.260; 95%CI, 1.591-6.680; *P*= 0.001) (**Supplementary Table 1**). Generally, selection of the therapy method was unaffected by the characteristics of the patients. To further investigate the correlation between OS and other parameters, univariate analysis was performed. As shown in **Supplementary Table 2**, tumor location (*P*< 0.001), therapy methods (*P*= 0.003) and AJCC stage (*P*< 0.001) were statistically significant predictors of OS. To determine the respective effect of these factors, multivariate analysis was applied to avoid interference. The *Cox* proportional-hazards model revealed that therapy methods (*P*< 0.001), tumor location (*P*= 0.02) and AJCC stage (*P*< 0.001) were independent prognostic predictors for OS in LS-SCLC patients (**Supplementary Table 2**). Increased AJCC stage was a significant risk factor for SCLC patients (stage II: HR, 2.182; 95%CI, 1.505-3.163; *P*< 0.001; stage III: HR, 2.174; 95%CI, 1.490-3.171; *P*< 0.001). Chemotherapy-plus-surgery treatment was associated with a favorable prognostic factor for SCLC (HR, 0.521; 95%CI, 0.384-0.706; *P*< 0.001).

### Survival Analysis

**Figure 2:**
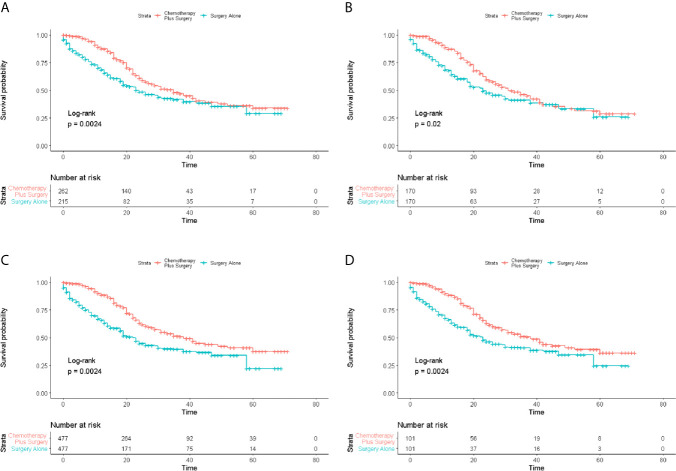
Survival analysis of overall survival (OS) for patients with limited-stage SCLC receiving chemotherapy-plus-surgery or surgery-alone treatment. **(A)** Kaplan-Meier analysis of OS before matching; **(B)** Kaplan-Meier analysis of OS after matching; **(C)** Kaplan-Meier analysis of OS after IPTW analysis; **(D)** Kaplan-Meier analysis of OS after Overlap Weighting analysis.

The 1-, 2-, 3-, 4- and 5-year OS rates of patents in the two groups are shown in **Table 2**. The median OS of the patients in surgery-plus-chemotherapy group was 35 months and was significantly better than that of surgery-alone patients (*P*= 0.002). The unmatched 1-, 3- and 5-year OS rate was 68.5%, 41.8% and 29.1% for surgery-alone group *vs.* 87.8%, 48.0% and 34.1% for surgery-plus-chemotherapy group, respectively. PSM, IPTW and Overlap Weighting analysis revealed the similar results (**Table 2**). The Kaplan–Meier OS survival curves are illustrated in **Figure 2**.

**Table 2:** Overall survival (%) of SCLC patients receiving chemotherapy plus surgery *versus* surgery alone.

Year	Unmatched (95% CI)		Matched (95% CI)		IPTW (95% CI)		Overlap Weighting (95% CI)	
	Chemotherapy Plus Surgery	Surgery Alone	Chemotherapy Plus Surgery	Surgery Alone	Chemotherapy Plus Surgery	Surgery Alone	Chemotherapy Plus Surgery	Surgery Alone
1	87.8(83.6-92.1)	68.5(62.3-75.4)	88.6(83.6-93.9)	67.9(60.9-75.8)	88.7(85.8-91.8)	65.8(61.5-70.4)	88.4(81.9-95.3)	66.3(57.3-76.7)
2	60.8(54.3-68.2)	48.5(41.5-56.7)	63.9(56.0-72.9)	47.6(39.8-56.9)	63.2(58.4-68.5)	44.7(40.0-50.1)	63.0(52.9-74.9)	46.2(36.4-58.5)
3	48.0(41.0-56.3)	41.8(34.6-50.4)	52.3(43.8-62.5)	41.3(33.4-51.0)	51.5(46.2-57.4)	39.6(34.8-45.1)	51.0(40.3-64.7)	40.9(31.1-53.8)
4	39.2(31.7-48.5)	35.5(28.0-45.0)	42.7(33.5-54.4)	33.4(25.1-44.4)	43.9(38.2-50.4)	34.1(29.0-40.0)	42.8(31.4-58.3)	34.5(24.3-49.0)
5	31.4(26.1-44.5)	29.1(20.2-41.9)	35.6(25.8-49.1)	26.0(16.6-40.8)	37.5(31.3-45.0)	22.1(16.1-30.5)	36.1(24.1-54.2)	24.5(12.9-46.7)

IPTW, inverse probability of treatment weight.

**Supplementary Table 3** shows the results of CSS. The median time of CSS was 45 months for surgery-alone group *vs.* 58 months for surgery-plus-chemotherapy group. However, there was no significant improvement in CSS by receiving surgery plus chemotherapy in the unmatched cohort (*P*= 0.08). The unmatched 1-, 3- and 5-year OS rate was 77.4%, 57.2% and 47.3% for surgery-alone group *vs.* 91.0%, 56.9% and 46.9% for surgery-plus-chemotherapy group, respectively. PSM, IPTW and Overlap Weighting analysis revealed the similar results (**Supplementary Table 3**). The Kaplan–Meier survival curves of CSS are illustrated in **Supplementary Figure 2**.

### Stratified Analysis

**Figure 3:**
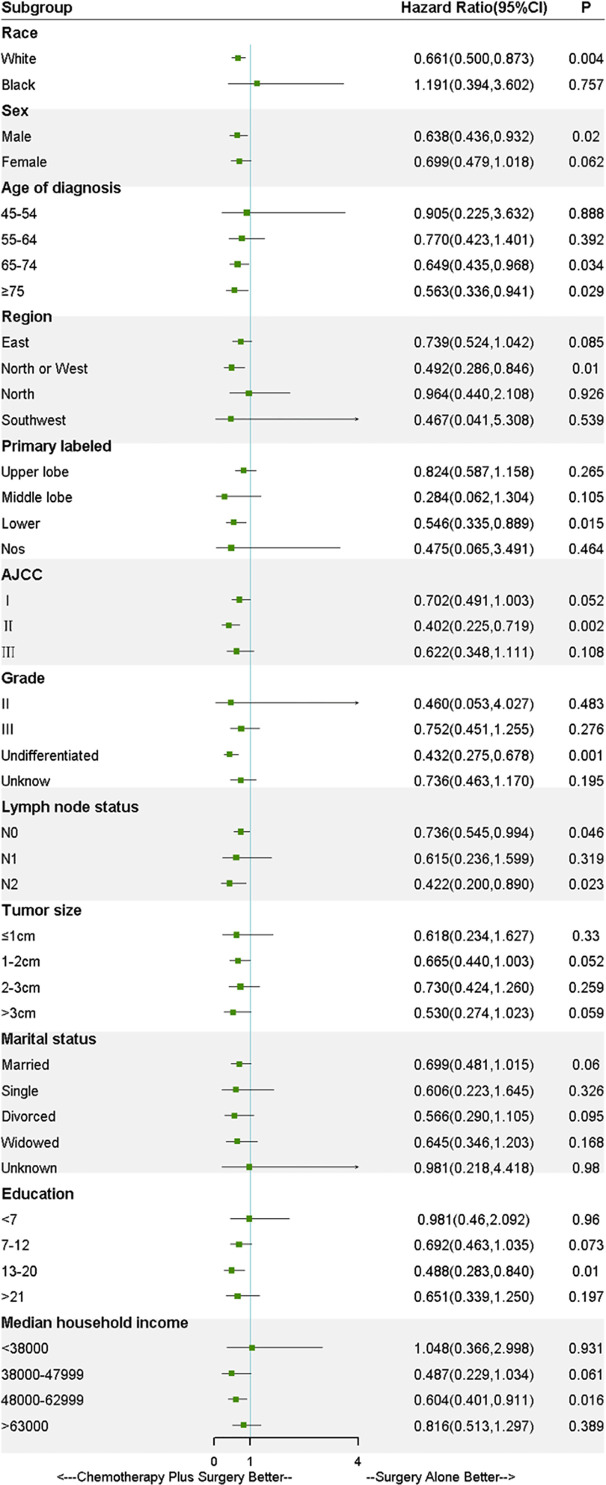
Forest plot of the stratified analysis.

As shown in **Figure 3**, to further explore the effect of treatments on the survival in LS-SCLC patients, all covariates were controlled in the stratified analysis. The result of stratified analysis revealed that benefit in OS was across all subgroups in chemotherapy-plus-surgery treatment compared with surgery-alone treatment, except for the black race and median household income <380000 USD/year. However, no significant difference was found in the black race (HR, 1.191; 95%CI, 0.394-3.602; *P*= 0.76) and median household income <380000 USD/year (HR, 1.048; 95%CI, 0.366-2.998; *P*= 0.93) between the two treatment groups. Stratified patients based on lymph node status, as illustrated in **Figure 3**, significant difference was found in N0 (HR, 0.736; 95%CI, 0.545-0.994; *P*= 0.046) and N2 (HR, 0.422; 95%CI, 0.200-0.890; *P*= 0.023) subgroups. Although no significant difference was found in N1 (HR, 0.615; 95%CI, 0.236-1.599; *P*= 0.319) subgroup, a tendency for improvement of OS was showed for the patients received surgery plus chemotherapy. Of note, survival analysis of patients based on AJCC stage stratification showed that OS of the unmatched patients with stage I (*P*= 0.049) and II (*P*= 0.001) SCLC who received surgery-plus-chemotherapy treatment was significantly better than that of surgery-alone patients. PSM, IPTW and Overlap Weighting analysis revealed the similar results (**Supplementary Figures 3** and **4**). As for patients with stage III SCLC, however, no significant difference in survival was observed (*P* = 0.10) (**Supplementary Figure 5**).

## Discussion

This cohort study was conducted utilizing retrospective data from the SEER database, comparing the survival outcomes of LS-SCLC patients who received either surgery-plus-chemotherapy treatment or surgery-alone treatment. In addition, it’s the first cohort study to compare survival time between LS-SCLC patients who received the two different treatment methods. By using multivariable analyses and PSM method to eliminate the selection bias, our results consistently demonstrated that surgery-plus-chemotherapy treatment significantly improved the median OS of LS-SCLC patients as compared with surgery alone. The result of stratified analysis based on AJCC stage showed that adjuvant chemotherapy was necessary after surgical resection for patients with LS-SCLC. Unlike the case with NSCLC, surgery-plus-chemotherapy treatment is recommended for stage I SCLC patients, and can be used to guide the management of LS-SCLC patients.

Given the greater proportion (85%) of NSCLC in all lung cancer cases (8), the treatment of NSCLC has been focused in most previous prospective and retrospective studies (9–12). Generally, it is recommended that adjuvant chemotherapy following resection for patients with stage II and IIIA NSCLC is required, but not for stage I patients (6). As for SCLC, the standard treatment for LS-SCLC patients is to combine platinum-based chemotherapy with thoracic radiation, but the prognosis tends to be modest (13, 14). Therefore, effective therapeutic strategy for LS-SCLC remains an unmet goal. As lymph node or blood metastasis usually occurs in early-stage SCLC, fewer than 5% SCLC patients were initially diagnosed at stage T1-2N0M0 (15, 16). It is often difficult to detect multiple tiny metastatic lesions in the early stage, and therefore multimodality treatment may be necessary for LS-SCLC patients. A growing number of studies conclude that survival time of the LS-SCLC patients can significantly longer after surgical treatment (17–23). Major oncology groups also favor guidelines, which support surgical resection should be an important part of the multimodality management for stage I SCLC patients (24). Two clinical trials in the 1990s (25, 26) demonstrated that surgical resection combined with chemotherapy was an effective used strategy for stage I-II SCLC patients. However, the two clinical trials included only a limited number of patients. Shepherd et al. also reported that surgery with adjuvant chemotherapy could significantly improve survival time and prognosis of patients with stage I SCLC (27). However, not only the sample size of the study was small, but also this study couldn’t avoid the patient selection bias and lack a control group for comparison purposes. Although Yang et al. demonstrated that adjuvant chemotherapy after surgery resulted in prolonged survival based on a larger sample size, this study only focused on patients with pT1-2N0M0 SCLC (28).

In this study, more patients were selected for statistical analysis to further verify the previous conclusions from the SEER database. In addition, this is the largest cohort study to evaluate whether chemotherapy is suitable for all patients with LS-SCLC who received surgical resection. In this study, we identified a total of 477 SCLC patients who fulfilled the eligibility criteria between 2004 and 2015. Survival analysis of our study revealed that both OS and CSS were improved in patients receiving surgery-plus-chemotherapy treatment as compared with surgery-alone treatment. Especially, surgery-plus-chemotherapy treatment significantly improved OS as compared with surgery-alone treatment. In stratified analysis, compared with surgery-alone treatment, surgery plus chemotherapy was beneficial to stage I-II SCLC patients, whereas no significant difference in survival was observed in stage III SCLC patients. This result may be explained by the small sample size of patients. Nearly two-thirds of the patients in our study were diagnosed with AJCC stage I, and only 80 patients (16.8%) and 77 patients (16.1%) were diagnosed with AJCC stage II and III, respectively. This result is consistent with the prior study in early-stage SCLC patients (28), further demonstrating that surgery-plus-chemotherapy treatment is beneficial to stage I SCLC patients, which is different from the NSCLC treatment. Besides, it is wildly accepted that lymph node metastasis is an independent risk factor for SCLC patients. It was obviously revealed that a tendency for improvement of OS was across N0, N1 and N2 subgroups in chemotherapy-plus-surgery treatment compared with surgery-alone treatment. Due to the relatively small sample size of patients, however, no significant difference was found in N1 (*P*= 0.319) subgroup.

To our best knowledge, this is the largest cohort study to compare survival time of two different treatment methods in LS-SCLC patients. Besides, we used IPTW, PSM and overlap weighting methods to minimize the effect of observed confounders efficiently, thus guaranteeing the reliability of the results. Nevertheless, our study also has certain limitations. First, this study is retrospective, therefore inherent selection bias is inevitable. Second, due to the sample size of this study was comparatively small, especially in subgroup analysis, our ability may be limited to identify the patients who would benefit more from surgery-plus-chemotherapy treatment. Besides, the SEER database lacks essential clinical details, such as description of the chemotherapy drugs used and the baseline lung function, knowing that these details may have impact on patient prognosis, but not significantly. Finally, our study mainly focused on the survival outcome of surgery plus chemotherapy vs. surgery alone in patients with LS-SCLC, thus the patients who received radiotherapy including those received prophylactic cranial irradiation (PCI) were completely excluded. Several previous studies have pointed out that PCI can improve the prognosis of SCLC patients, however, nearly 70% patients in our study were stage I SCLC, the efficacy of PCI in this population may be unsatisfactory as the study reported by Xu et al. (29). Hence, based on the previous studies combined with clinical practice, the conclusion in our study was reliable. Next, we have conducted a new research focusing on the effectiveness of radiotherapy or chemoradiotherapy combined with surgery for LS-SCLC patients.

In conclusion, our retrospective analysis of the SEER database revealed that surgery plus chemotherapy was associated with longer survival time than surgery alone in patients with LSSCLC, especially those with stage I and II SCLC. Prospective studies are therefore needed to confirm our findings and conclusions.

## Data Availability Statement

Publicly available datasets were analyzed in this study. This data can be found here: https://seer.cancer.gov/data/.

## Ethics Statement

The studies involving human participants were reviewed and approved by Shanghai Tenth People’s Hospital. Written informed consent was not provided because Patients from the Surveillance, Epidemiology, and End Results (SEER) database had previously consented to participate in any scientific research worldwide.

## Author Contributions

ML and CD contributed to conception and design of the study. ZG performed data extraction. YZ and PY performed the statistical analysis. PY wrote the main manuscripts. All authors contributed to the article and approved the submitted version.

## Funding

This work was financially supported by the National Natural Science Foundation of China (No. 81802262, 31770131, 81473469); the Fundamental Research Funds for the Central Universities (No. 22120180584); Shanghai Tenth Hospital’s Improvement Plan for NSFC (No. 04.03.17.032, 04.01.18. 048, SYGZRPY2017014); Shanghai Municipal Health Planning Commission Project (No. ZHYY-ZXYJHZX-201607); Shanghai Shen Kang Hospital Development Center Plan (SHDC12018119) and Scientific Research Projects of Shanghai Municipal Commission of Health and Family Planning (201840056).

## Conflict of Interest

The authors declare that the research was conducted in the absence of any commercial or financial relationships that could be construed as a potential conflict of interest.

## Supplementary Material

Supplementary Figure 1 Plot of standardized mean differences (SMD) of baseline covariates. **(A)** SMD after propensity score matching; **(B)** SMD after IPTW analysis; **(C)** SMD after Overlap Weighting analysis.

Supplementary Figure 2 Survival analyses of cause-specific survival (CSS) for patients with limited-stage SCLC receiving chemotherapy-plus-surgery or surgery-alone treatment. **(A)** Kaplan-Meier analysis of CSS before matching; **(B)** Kaplan-Meier analysis of CSS before matching; **(C)** Kaplan-Meier analysis of CSS after IPTW analysis; **(D)** Kaplan-Meier analysis of CSS after Overlap Weighting analysis.

Supplementary Figure 3 Survival analyses of overall survival (OS) for patients with AJCC stage I SCLC receiving chemotherapy-plus-surgery or surgery-alone treatment. **(A)** Kaplan-Meier analysis of OS before matching; **(B)** Kaplan-Meier analysis of OS after matching; **(C)** Kaplan-Meier analysis of OS after IPTW analysis; **(D)** Kaplan-Meier analysis of OS after Overlap Weighting analysis.

Supplementary Figure 4 Survival analyses of overall survival (OS) for patients with AJCC stage II SCLC receiving chemotherapy-plus-surgery or surgery-alone treatment. **(A)** Kaplan-Meier analysis of OS before matching; **(B)** Kaplan-Meier analysis of OS after matching; **(C)** Kaplan-Meier analysis of OS after IPTW analysis; **(D)** Kaplan-Meier analysis of OS after Overlap Weighting analysis.

Supplementary Figure 5 Survival analyses of overall survival (OS) for patients with AJCC stage III SCLC receiving chemotherapy-plus-surgery or surgery-alone treatment. **(A)** Kaplan-Meier analysis of OS before matching; **(B)** Kaplan-Meier analysis of OS after matching; **(C)** Kaplan-Meier analysis of OS after IPTW analysis; **(D)** Kaplan-Meier analysis of OS after Overlap Weighting analysis.

## Abbreviations

LS-SCLC, limited-stage small-cell lung cancer; NSCLC, non-small-cell lung carcinoma; OS, overall survival; CSS, cause-specific survival; SEER, Surveillance, Epidemiology, and End Results; AJCC, American Joint Committee on Cancer; OR, odds ratio; HR, hazard ratio; CI, confidence interval; PS, propensity score; PSM, propensity score matching; IPTW, inverse probability of treatment weighting; PCI, prophylactic cranial irradiation.

## References

[1] Shao C He J Kachroo S Jin F. Chemotherapy Treatments, Costs of Care, and Survival for Patients Diagnosed With Small Cell Lung Cancer: A Seer-Medicare Study. Cancer Med (2019) 8(18):7613–22. 10.1002/cam4.2626 PMC6912057 31668011

[2] Sabari JK Lok BH Laird JH Poirier JT Rudin CM. Unravelling the Biology of SCLC: Implications for Therapy. Nat Rev Clin Oncol (2017) 14(9):549–61. 10.1038/nrclinonc.2017.71 PMC5843484 28534531

[3] Kalemkerian GP Akerley W Bogner P Borghaei H Chow LQ Downey RJ. Small Cell Lung Cancer. J Natl Compr Cancer Netw (2013) 11(1):78–98. 10.6004/jnccn.2013.0011 PMC3715060 23307984

[4] Siegel R Ma J Zou Z Jemal A. Cancer Statistics, 2014. CA Cancer J Clin (2014) 64(1):9–29. 10.3322/caac.21208 24399786

[5] Puma F Urbani M Santoprete S Ricci F Sanguinetti A Vinci D. The Role of Surgery in the Treatment of Small Cell Lung Cancer. Minerva Endocrinol (2001) 26(4):247–53. 11782710

[6] Duma N Santana-Davila R Molina JR. Non-Small Cell Lung Cancer: Epidemiology, Screening, Diagnosis, and Treatment. Mayo Clin Proc (2019) 94(8):1623–40. 10.1016/j.mayocp.2019.01.013 31378236

[7] Cronin KA Ries LAG Edwards BK. The Surveillance, Epidemiology, and End Results (SEER) Program of the National Cancer Institute. Cancer (2014) 120 Suppl 23:3755–7. 10.1002/cncr.29049 25412387

[8] Lu T Yang X Huang Y Zhao M Li M Ma K. Trends in the Incidence, Treatment, and Survival of Patients With Lung Cancer in the Last Four Decades. Cancer Manage Res (2019) 11:943–53. 10.2147/CMAR.S187317 PMC6345192 30718965

[9] Ning Y Bao M Yan X Xie D Jiang G. Surgery for Advanced Non-Small Cell Lung Cancer Patient After Epidermal Growth Factor Receptor Tyrosine Kinase Inhibitor Neoadjuvant Therapy. Ann Transl Med (2018) 6(20):407. 10.21037/atm.2018.10.06 30498734 PMC6230859

[10] Schreiner W Gavrychenkova S Dudek W Rieker RJ Lettmaier S Fietkau R. Pathologic Complete Response After Induction Therapy-the Role of Surgery in Stage IIIA/B Locally Advanced Non-Small Cell Lung Cancer. J Thorac Dis (2018) 10(5):2795–803. 10.21037/jtd.2018.05.68 PMC6006117 29997942

[11] Gonzalez-Rivas D Fieira E Delgado M Mendez L Fernandez R de la Torre M. Is Uniportal Thoracoscopic Surgery a Feasible Approach for Advanced Stages of non-Small Cell Lung Cancer? J Thorac Dis (2014) 6(6):641–8. 10.3978/j.issn.2072-1439.2014.05.17 PMC4073401 24976985

[12] Uramoto H Akiyama H Nakajima Y Kinoshita H Inoue T Kurimoto F. The Long-Term Outcomes of Induction Chemoradiotherapy Followed by Surgery for Locally Advanced non-Small Cell Lung Cancer. Case Rep Oncol (2014) 7(3):700–10. 10.1159/000368598 PMC4255996 25493083

[13] Faivre-Finn C Snee M Ashcroft L Appel W Barlesi F Bhatnagar A. Concurrent Once-Daily Versus Twice-Daily Chemoradiotherapy in Patients With Limited-Stage Small-Cell Lung Cancer (CONVERT): An Open-Label, Phase 3, Randomised, Superiority Trial. Lancet Oncol (2017) 18(8):1116–25. 10.1016/S1470-2045(17)30318-2 PMC5555437 28642008

[14] Grønberg BH Killingberg KT Fløtten Ø Brustugun OT Hornslien K Madebo T. High-Dose Versus Standard-Dose Twice-Daily Thoracic Radiotherapy for Patients With Limited Stage Small-Cell Lung Cancer: An Open-Label, Randomised, Phase 2 Trial. Lancet Oncol (2021) 22(3):321–31. 10.1016/S1470-2045(20)30742-7 33662285

[15] Lally BE Urbanic JJ Blackstock AW Miller AA Perry MC. Small Cell Lung Cancer: Have We Made Any Progress Over the Last 25 Years? Oncologist (2007) 12(9):1096–104. 10.1634/theoncologist.12-9-1096 17914079

[16] Takei H Kondo H Miyaoka E Asamura H Yoshino I Date H. Surgery for Small Cell Lung Cancer: A Retrospective Analysis of 243 Patients From Japanese Lung Cancer Registry in 2004. J Thorac Oncol (2014) 9(8):1140–5. 10.1097/JTO.0000000000000226 25157766

[17] Che K Shen H Qu X Pang Z Jiang Y Liu S. Survival Outcomes for Patients With Surgical and Non-Surgical Treatments in Stages I-III Small-Cell Lung Cancer. J Cancer (2018) 9(8):1421–9. 10.7150/jca.23583 PMC5929087 29721052

[18] Brock MV Hooker CM Syphard JE Westra W Xu L Alberg AJ. Surgical Resection of Limited Disease Small Cell Lung Cancer in the New Era of Platinum Chemotherapy: Its Time has Come. J Thorac Cardiovasc Surg (2005) 129(1):64–72. 10.1016/j.jtcvs.2004.08.022 15632826

[19] Lewiński T Zuławski M Turski C Pietraszek A. Small Cell Lung Cancer I–III A: Cytoreductive Chemotherapy Followed by Resection With Continuation of Chemotherapy. Eur J Cardiothorac Surg (2001) 20(2):391–8. 10.1016/S1010-7940(01)00787-4 11463563

[20] Shields TW Higgins GA Matthews MJ Keehn RJ. Surgical Resection in the Management of Small Cell Carcinoma of the Lung. J Thorac Cardiovasc Surg (1982) 84(4):481–8. 10.1016/S0022-5223(19)38975-5 6289013

[21] Shepherd FA Ginsberg RJ Patterson GA Evans WK Feld R. A Prospective Study of Adjuvant Surgical Resection After Chemotherapy for Limited Small Cell Lung Cancer. A University of Toronto Lung Oncology Group Study. J Thorac Cardiovasc Surg (1989) 97(2):177–86. 10.1016/S0022-5223(19)35322-X 2536868

[22] Shepherd FA Ginsberg RJ Feld R Evans WK Johansen E. Surgical Treatment for Limited Small-Cell Lung Cancer. The University of Toronto Lung Oncology Group Experience. J Thorac Cardiovasc Surg (1991) 101(3):385–93. 10.1016/S0022-5223(19)36720-0 1847981

[23] Lüchtenborg M Riaz SP Lim E Page R Baldwin DR Jakobsen E. Survival of Patients With Small Cell Lung Cancer Undergoing Lung Resection in England, 1998-2009. Thorax (2014) 69(3):269–73. 10.1136/thoraxjnl-2013-203884 PMC3932952 24172710

[24] Früh M De Ruysscher D Popat S Crinò L Peters S Felip E. Small-Cell Lung Cancer (SCLC): ESMO Clinical Practice Guidelines for Diagnosis, Treatment and Follow-Up. Ann Oncol (2013) 24 Suppl 6:vi99–105. 10.1093/annonc/mdt178 23813929

[25] Hara N Ohta M Ichinose Y Motohiro A Kuda T Asoh H. Influence of Surgical Resection Before and After Chemotherapy on Survival in Small Cell Lung Cancer. J Surg Oncol (1991) 47(1):53–61. 10.1002/jso.2930470112 1850811

[26] Shibayama T Hiyama J Ueoka H Tabata M Segawa Y Gemba K. Induction Chemotherapy Followed by Adjuvant Surgery (IC-AS) in Patients With Stage I-II Small Cell Lung Cancer (SCLC). Gan To Kagaku Ryoho (1995) 22(13):1953–8. 7487126

[27] Shepherd FA Evans WK Feld R Young V Patterson GA Ginsberg R. Adjuvant Chemotherapy Following Surgical Resection for Small-Cell Carcinoma of the Lung. J Clin Oncol (1988) 6(5):832–8. 10.1200/JCO.1988.6.5.832 2835443

[28] Yang C-FJ Chan DY Speicher PJ Gulack BC Wang X Hartwig MG. Role of Adjuvant Therapy in a Population-Based Cohort of Patients With Early-Stage Small-Cell Lung Cancer. J Clin Oncol (2016) 34(10):1057–64. 10.1200/JCO.2015.63.8171 PMC4933132 26786925

[29] Xu J Yang H Fu X Jin B Lou Y Zhang Y. Prophylactic Cranial Irradiation for Patients With Surgically Resected Small Cell Lung Cancer. J Thorac Oncol (2017) 12(2):347–53. 10.1016/j.jtho.2016.09.133 27725211

