# Supplementary figures and images for: Surgery Plus Chemotherapy Versus Surgery Alone for Limited-Stage Small-Cell Lung Cancer: A Population-Based Survival Outcome Analysis
Source: Front Oncol. 2021 May 17;11:676598. doi: 10.3389/fonc.2021.676598 (PMC8165284; doi:10.3389/fonc.2021.676598)

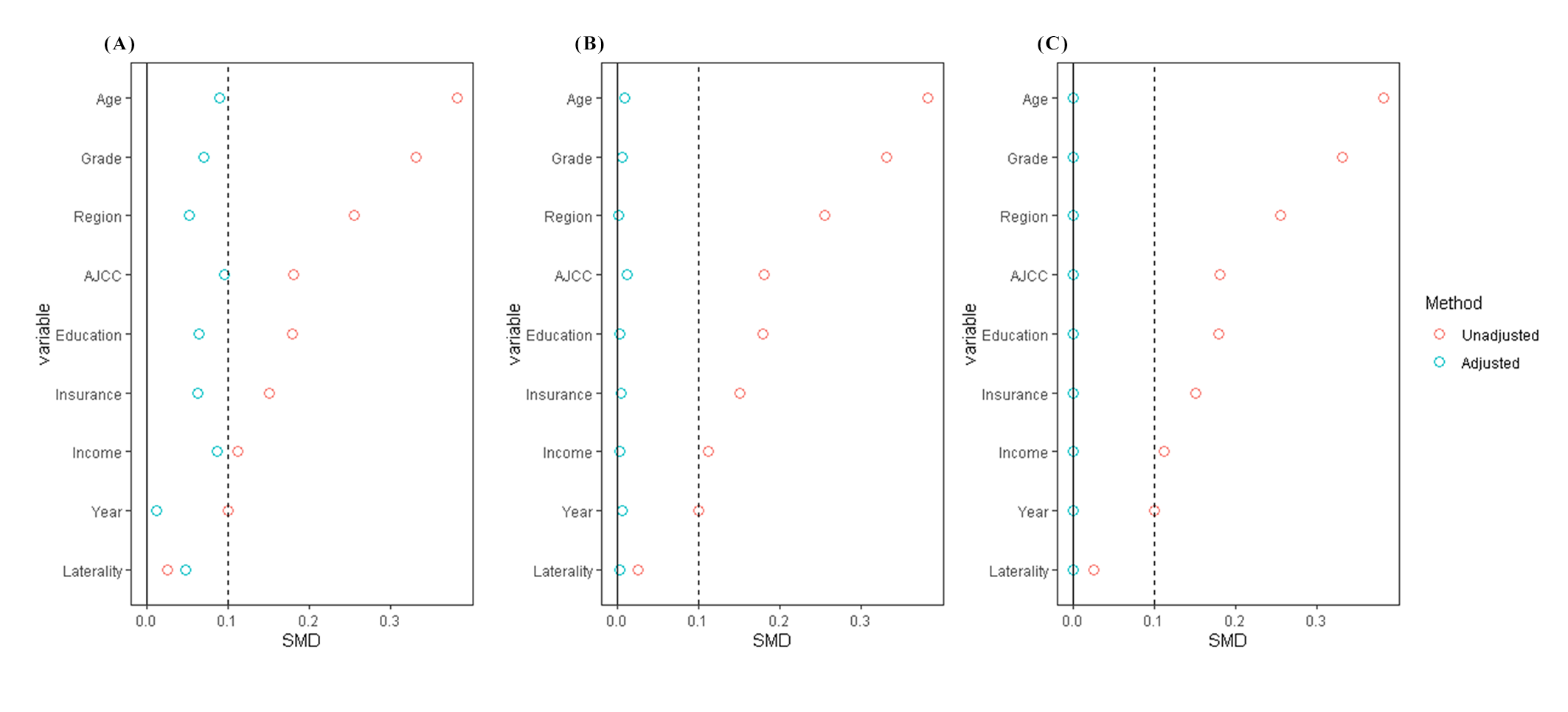

Supplement: Supplementary Figure 1 — Plot of standardized mean differences (SMD) of baseline covariates. (A) SMD after propensity score matching; (B) SMD after IPTW analysis; (C) SMD after Overlap Weighting analysis. [file Image_1.tif]

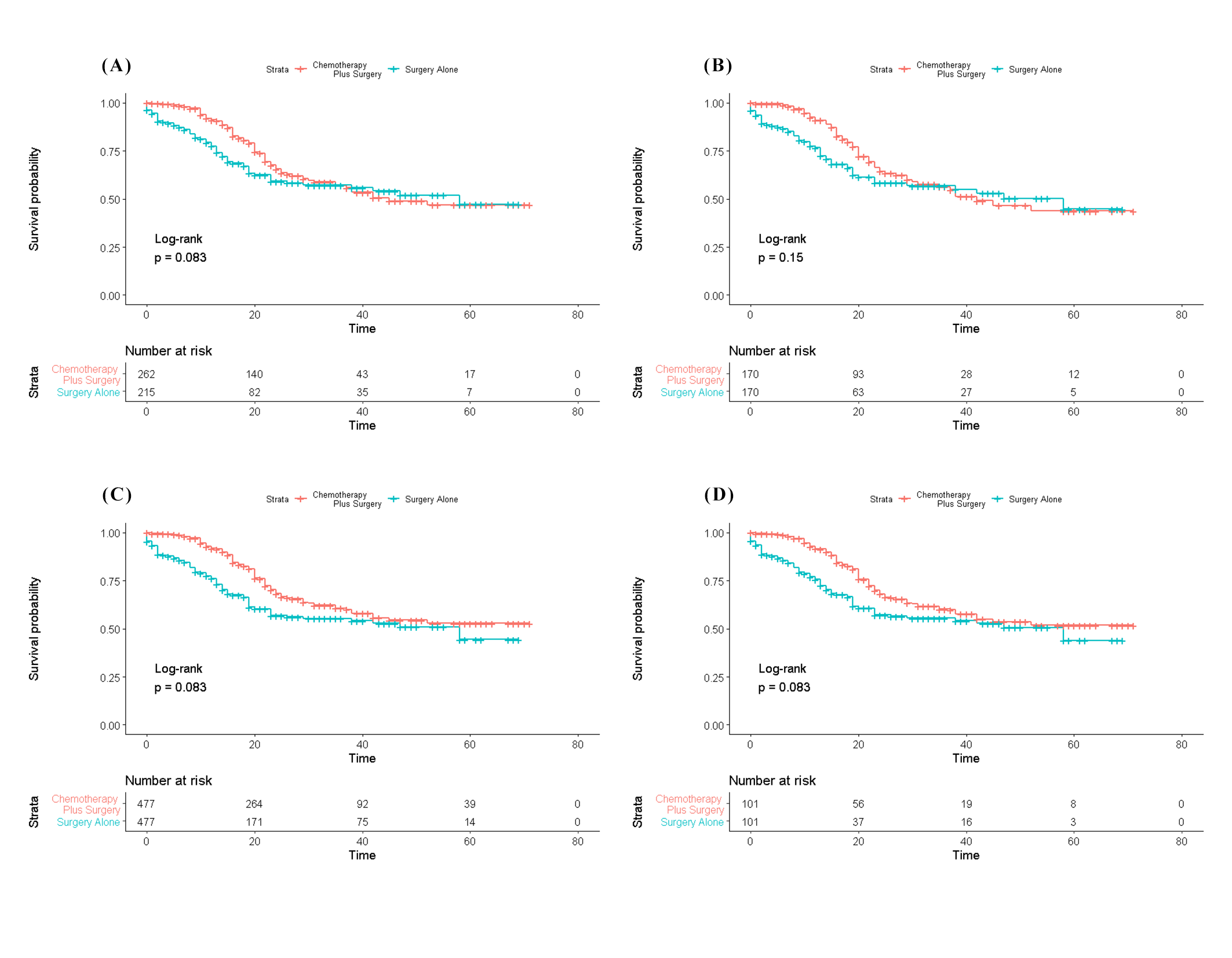

Supplement: Supplementary Figure 2 — Survival analyses of cause-specific survival (CSS) for patients with limited-stage SCLC receiving chemotherapy-plus-surgery or surgery-alone treatment. (A) Kaplan-Meier analysis of CSS before matching; (B) Kaplan-Meier analysis of CSS before matching; (C) Kaplan-Meier analysis of CSS after IPTW analysis; (D) Kaplan-Meier analysis of CSS after Overlap Weighting analysis. [file Image_2.tif]

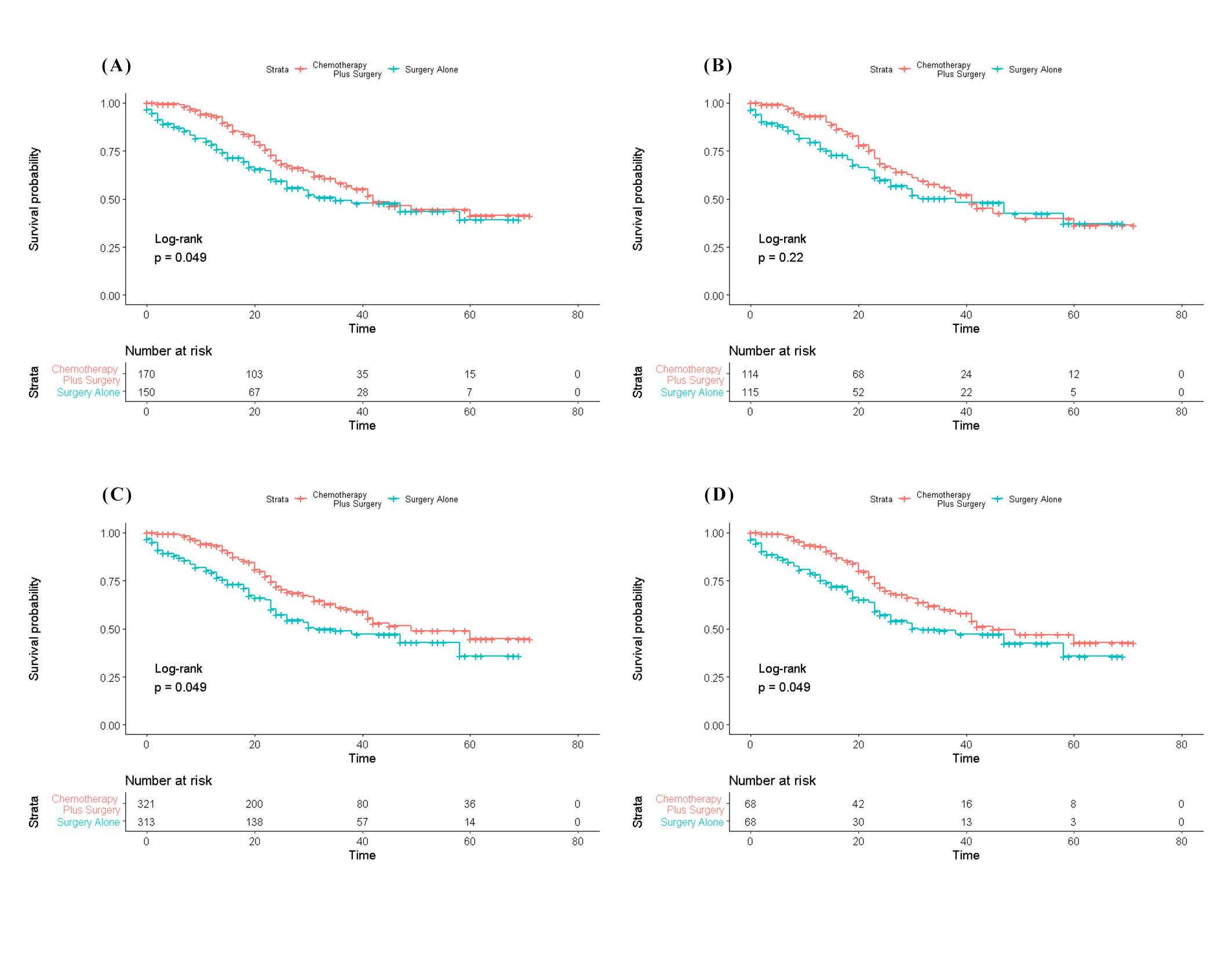

Supplement: Supplementary Figure 3 — Survival analyses of overall survival (OS) for patients with AJCC stage I SCLC receiving chemotherapy-plus-surgery or surgery-alone treatment. (A) Kaplan-Meier analysis of OS before matching; (B) Kaplan-Meier analysis of OS after matching; (C) Kaplan-Meier analysis of OS after IPTW analysis; (D) Kaplan-Meier analysis of OS after Overlap Weighting analysis. [file Image_3.tif]

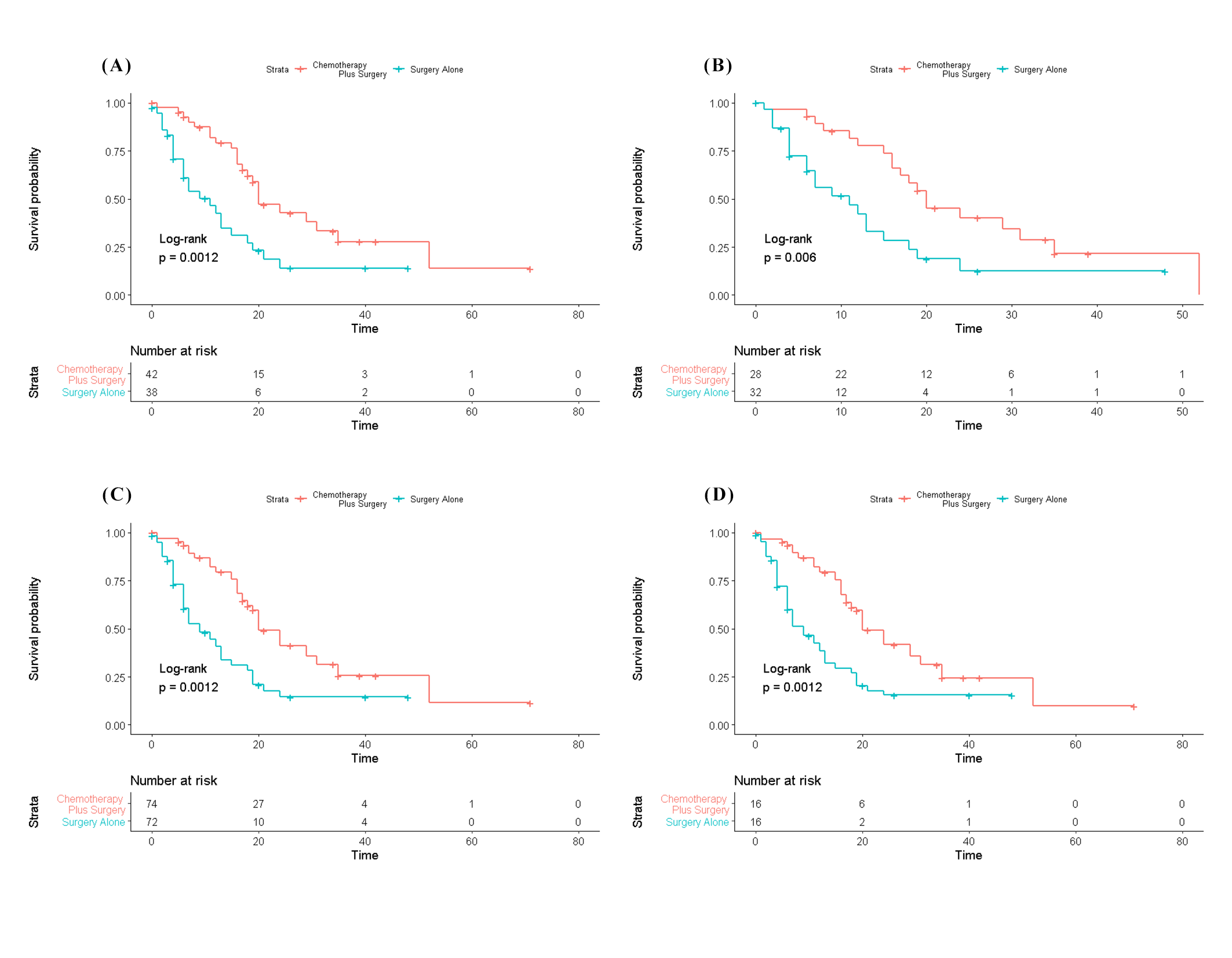

Supplement: Supplementary Figure 4 — Survival analyses of overall survival (OS) for patients with AJCC stage II SCLC receiving chemotherapy-plus-surgery or surgery-alone treatment. (A) Kaplan-Meier analysis of OS before matching; (B) Kaplan-Meier analysis of OS after matching; (C) Kaplan-Meier analysis of OS after IPTW analysis; (D) Kaplan-Meier analysis of OS after Overlap Weighting analysis. [file Image_4.tif]

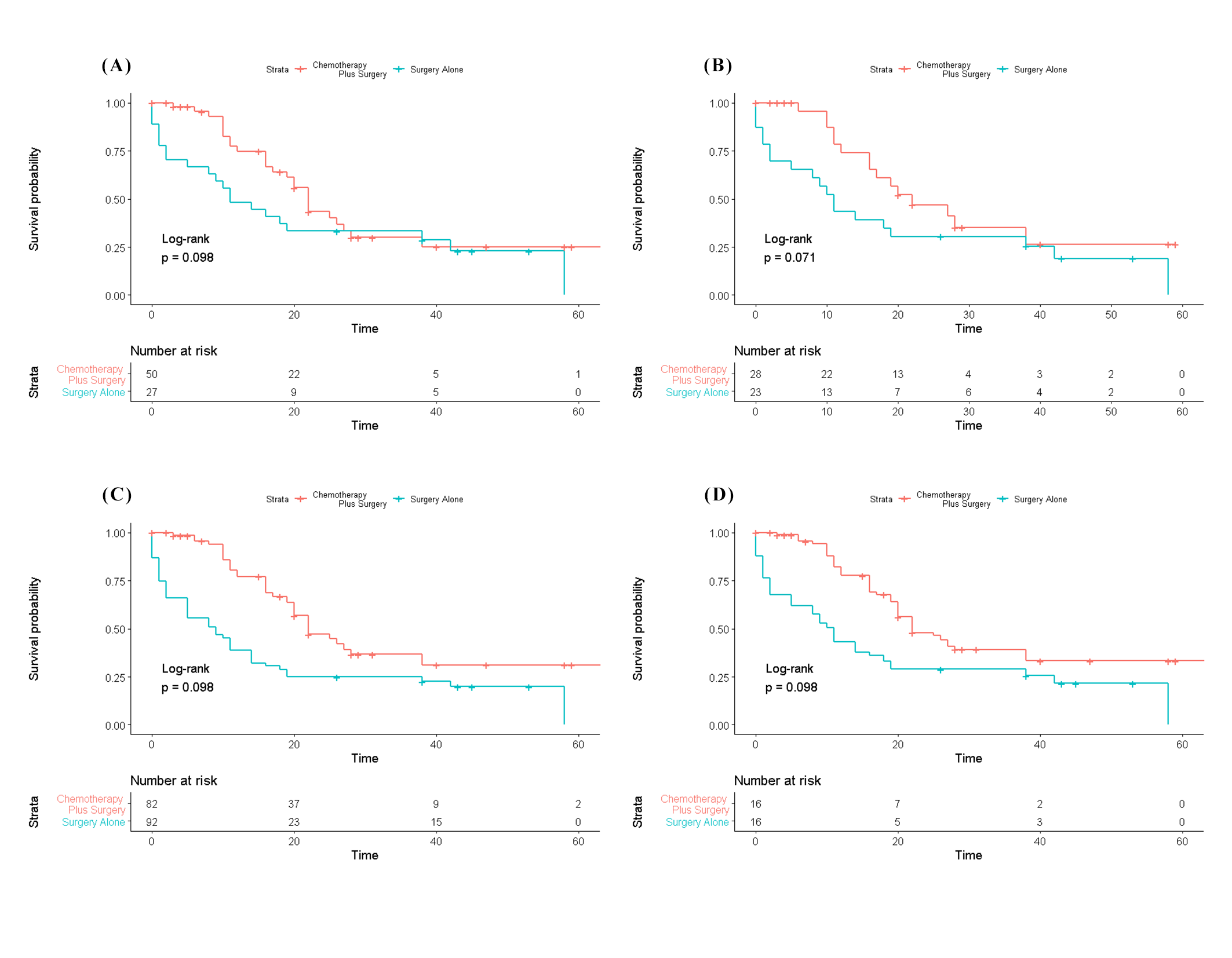

Supplement: Supplementary Figure 5 — Survival analyses of overall survival (OS) for patients with AJCC stage III SCLC receiving chemotherapy-plus-surgery or surgery-alone treatment. (A) Kaplan-Meier analysis of OS before matching; (B) Kaplan-Meier analysis of OS after matching; (C) Kaplan-Meier analysis of OS after IPTW analysis; (D) Kaplan-Meier analysis of OS after Overlap Weighting analysis. [file Image_5.tif]
