# Supplementary material for: Surgery Plus Chemotherapy Versus Surgery Alone for Limited-Stage Small-Cell Lung Cancer: A Population-Based Survival Outcome Analysis
Source: Front Oncol. 2021 May 17;11:676598. doi: 10.3389/fonc.2021.676598 (PMC8165284; doi:10.3389/fonc.2021.676598)
Supplement: Supplementary file 6 [file DataSheet_1.docx]

**Supplementary Table**

**Supplementary Table 1. Propensity Modeling of Receipt of Chemotherapy Plus Surgery.**

**Supplementary Table 2. Univariate and Multivariate Analyses of Overall Survival for Limited-stage Small Cell Lung Cancer Using Cox Proportional Hazards Model.**

**Supplementary Table 3. Cancer-specific Survival (%) of SCLC Patients Receiving Chemotherapy Plus Surgery *versus* Surgery Alone.**

| **Supplementary Table 1. Propensity Modeling of Receipt of Chemotherapy Plus Surgery.** | | | |
| --- | --- | --- | --- |
| **Characteristics** | **OR** | **95% CI** | ***P*** |
| Age of diagnosis |  |  |  |
| ≤44 | Reference |  |  |
| 45-54 | 2.254 | 0.090 to 56.6 | 0.62 |
| 55-64 | 2.422 | 0.106 to 55.375 | 0.58 |
| 65-74 | 1.561 | 0.70 to 34.886 | 0.78 |
| ≥75 | 4.231 | 0.185 to 96.932 | 0.37 |
| Region |  |  |  |
| East | Reference |  |  |
| North or West | 1.348 | 0.863 to 2.106 | 0.19 |
| North | 0.725 | 0.383 to 1.372 | 0.32 |
| Southwest | 0.314 | 0.078 to 1.256 | 0.10 |
| Primary labeled |  |  |  |
| Upper lobe | Reference |  |  |
| Middle lobe | 0.669 | 0.27 to 1.656 | 0.39 |
| Lower | 0.674 | 0.429 to 1.058 | 0.09 |
| Nos | 1.184 | 0.289 to 4.844 | 0.81 |
| Overlapping | 6.295 | 1.032 to 38.386 | 0.05 |
| AJCC |  |  |  |
| stage I | Reference |  |  |
| stage II | 1.027 | 0.601 to 1.753 | 0.92 |
| stage III | 0.536 | 0.300 to 0.985 | 0.04 |
| Grade |  |  |  |
| I | Reference |  |  |
| II | 1.186 | 0.151 to 9.316 | 0.87 |
| III | 0.401 | 0.074 to 2.172 | 0.29 |
| Undifferentiated | 0.275 | 0.051 to 1.498 | 0.14 |
| Unknow | 0.224 | 0.041 to 1.218 | 0.08 |
| Marital status |  |  |  |
| Married | Reference |  |  |
| Single | 3.260 | 1.591 to 6.680 | 0.001 |
| Divorced | 1.348 | 0.757 to 2.400 | 0.31 |
| Widowed | 1.083 | 0.625 to 1.877 | 0.78 |
| Unknown | 2.559 | 0.970 to 6.753 | 0.06 |
| Abbreviations: OR, Odds Ratio. | | | |

| **Supplementary Table 2. Univariate and Multivariate Analyses of Overall Survival for Limited-stage Small Cell Lung Cancer Using Cox Proportional Hazards Model.** | | | | | |
| --- | --- | --- | --- | --- | --- |
| **Variables** | **OS** | | | | |
|  | **Univariate** | |  | **Multivariate** | |
|  | **HR (95%CI)** | ***P*** |  | **HR (95%CI)** | ***P*** |
| Race |  | 0.94 |  |  | 0.98 |
| White | Reference |  |  | Reference |  |
| Black | 0.834 (0.476,1.462) | 0.53 |  | 0.883 (0.487,1.601) | 0.68 |
| Sex |  | 0.20 |  |  | 0.17 |
| Male | Reference |  |  | Reference |  |
| Female | 0.838 (0.642,1.095) |  |  | 0.805 (0.591,1.097) |  |
| Age of  diagnosis |  | 0.79 |  |  | 0.17 |
| ≤44 | Reference |  |  | Reference |  |
| 45-54 | 0.999 (0.125,7.992) | >0.99 |  | 0.445 (0.052,3.787) | 0.46 |
| 55-64 | 1.297 (0.178,9.432) | 0.80 |  | 0.629 (0.081,4.870) | 0.66 |
| 65-74 | 1.914 (0.267,13.742) | 0.52 |  | 0.947 (0.125,7.159) | 0.96 |
| ≥75 | 1.975 (0.274,14.257) | 0.50 |  | 0.824 (0.107,6.338) | 0.85 |
| Region |  | 0.52 |  |  | 0.69 |
| East | Reference |  |  | Reference |  |
| Northwest or West | 1.056 (0.770,1.448) | 0.73 |  | 0.930 (0.609,1.420) | 0.74 |
| North | 0.988 (0.648,1.505) | 0.95 |  | 1.090 (0.664,1.790) | 0.73 |
| Southwest | 0.484 (0.179,1.310) | 0.15 |  | 0.571 (0.202,1.592) | 0.28 |
| Primary labeled |  | <0.001 |  |  | 0.02 |
| Upper lobe | Reference |  |  | Reference |  |
| Middle lobe | 0.538 (0.252,1.151) | 0.11 |  | 0.665 (0.306,1.447) | 0.30 |
| Lower | 1.043 (0.775,1.403) | 0.78 |  | 1.054 (0.764,1.456) | 0.75 |
| Nos | 1.351 (0.553,3.301) | 0.51 |  | 1.271 (0.483,3.347) | 0.63 |
| Overlapping | 26.257 (7.901,87.261) | <0.001 |  | 9.957 (2.658,37.301) | 0.001 |
| AJCC |  | <0.001 |  |  | <0.001 |
| stage I | Reference |  |  | Reference |  |
| **Supplementary Table 2. Univariate and Multivariate Analyses of Overall Survival for Limited-stage Small Cell Lung Cancer Using Cox Proportional Hazards Model (continued).** | | | | | |
| **Variables** | **OS** | | | | |
|  | **Univariate** | |  | **Multivariate** | |
|  | **HR (95%CI)** | ***P*** |  | **HR (95%CI)** | ***P*** |
| stage II | 2.550 (1.818,3.575) | <0.001 |  | 2.182 (1.505,3.163) | <0.001 |
| stage III | 2.061 (1.471,2.889) | <0.001 |  | 2.174 (1.490,3.171) | <0.001 |
| Tumor size |  | 0.54 |  |  | 0.56 |
| ≤1cm | Reference |  |  | Reference |  |
| 1-2cm | 1.309 (0.780,2.198) | 0.31 |  | 1.402 (0.818,2.403) | 0.22 |
| 2-3cm | 1.327 (0.769,2.289) | 0.31 |  | 1.429 (0.804,2.542) | 0.22 |
| >3cm | 1.498 (0.868,2.584) | 0.15 |  | 1.217 (0.679,2.181) | 0.51 |
| Marital status |  | 0.24 |  |  | 0.29 |
| Married | Reference |  |  | Reference |  |
| Single | 1.310 (0.829,2.070) | 0.25 |  | 1.273 (0.748,2.168) | 0.37 |
| Divorced | 0.970 (0.663,1.418) | 0.88 |  | 1.008 (0.657,1.548) | 0.97 |
| Widowed | 1.348 (0.938,1.936) | 0.11 |  | 1.283 (0.853,1.930) | 0.23 |
| Unknown | 0.558 (0.260,1.198) | 0.13 |  | 0.463 (0.207,1.034) | 0.06 |
| Education |  | 0.19 |  |  | 0.17 |
| <7 | Reference |  |  | Reference |  |
| 7-12 | 1.537 (1.004,2.355) | 0.05 |  | 1.691 (1.028,2.782) | 0.04 |
| 12-20 | 1.229 (0.774,1.952) | 0.38 |  | 1.318 (0.737,2.358) | 0.35 |
| >21 | 1.450 (0.882,2.383) | 0.14 |  | 1.478 (0.712,3.066) | 0.29 |
| Median household  income  (dollar, in tens) |  | 0.53 |  |  | 0.73 |
| <38000 | Reference |  |  | Reference |  |
| 38000-47999 | 1.279 (0.693,2.361) | 0.43 |  | 1.275 (0.639,2.543) | 0.49 |
| 48000-62999 | 1.434 (0.817,2.518) | 0.21 |  | 1.393 (0.701,2.770) | 0.34 |
| **Supplementary Table 2. Univariate and Multivariate Analyses of Overall Survival for Limited-stage Small Cell Lung Cancer Using Cox Proportional Hazards Model (continued).** | | | | | |
| **Variables** | **OS** | | | | |
|  | **Univariate** | |  | **Multivariate** | |
|  | **HR (95%CI)** | ***P*** |  | **HR (95%CI)** | ***P*** |
| >63000 | 1.214 (0.684,2.153) | 0.51 |  | 1.218 (0.571,2.596) | 0.61 |
| Therapy method |  | 0.003 |  |  | <0.001 |
| Surgery alone | Reference |  |  | Reference |  |
| Chemotherapy  plus surgery | 0.666 (0.510,0.871) |  |  | 0.521 (0.384,0.706) |  |
| Abbreviations: OS, Overall Survival; HR, Hazard Ratio. | | | | | |

| **Supplementary Table 3. Cancer-specific Survival (%) of SCLC Patients Receiving Chemotherapy Plus Surgery *versus* Surgery Alone.** | | | | | | | | | | | |  |
| --- | --- | --- | --- | --- | --- | --- | --- | --- | --- | --- | --- | --- |
| **Year** | **Unmatched (95% CI)** | |  | **Matched (95% CI)** | |  | **IPTW (95% CI)** | |  | **Overlap Weighting (95% CI)** | | |
|  | **Chemotherapy Plus Surgery** | **Surgery Alone** |  | **Chemotherapy Plus Surgery** | **Surgery Alone** |  | **Chemotherapy Plus Surgery** | **Surgery Alone** |  | **Chemotherapy Plus Surgery** | **Surgery Alone** | |
| 1 | 91.0(87.3-94.9) | 77.4(71.5-83.7) |  | 92.3(88.1(96.8) | 76.6(70.0-83.8) |  | 91.8(90.3-95.3) | 76.1(72.1-80.4) |  | 91.6(86.0-97.7) | 75.7(67.2-85.3) | |
| 2 | 65.5(59.0-72.8) | 59.2(51.9-67.6) |  | 69.0(61.1-77.9) | 58.3(50.1-67.8) |  | 68.2(63.4-73.4) | 56.8(51.7-62.4) |  | 68.0(58.0-79.8) | 57.1(46.7-69.9) | |
| 3 | 56.9(49.7-65.1) | 57.2(49.7-65.9) |  | 61.0(52.4-71.1) | 56.9(48.6(66.6) |  | 60.7(55.4-66.5) | 55.2(50.0-61.0) |  | 60.2(49.3-73.6) | 55.5(44.9-68.6) | |
| 4 | 48.9(40.7-58.7) | 52.0(43.5-62.2) |  | 53.3(43.4-65.3) | 50.3(40.7-62.2) |  | 54.4(48.4-61.1) | 50.9(5.1-57.5) |  | 53.7(41.5-69.5) | 50.6(38.8-66.0) | |
| 5 | 46.9(38.3-57.3) | 47.3(36.5-61.2) |  | 50.5(40.1-63.5) | 44.7(32.7-61.2) |  | 52.7(46.5-59.8) | 44.4(36.8-53.6) |  | 51.8(39.1-68.6) | 43.9(28.7-67.1) | |
| Abbreviations: IPTW, inverse probability of treatment weight. | | | | | | | | | | | |  |
